# Analysis of Health Care Billing via Quantile Variable Selection Models

**DOI:** 10.3390/healthcare9101274

**Published:** 2021-09-27

**Authors:** Tahir Ekin, Paul Damien

**Affiliations:** 1 McCoy College of Business, Texas State University, San Marcos, TX 78666, USA; tahirekin@txstate.edu; 2 McCombs School of Business, University of Texas in Austin, Austin, TX 78712, USA

**Keywords:** health care fraud, medicare, quantile regression, upcoding, Bayesian information criterion

## Abstract

Fraudulent billing of health care insurance programs such as Medicare is in the billions of dollars. The extent of such overpayments remains an issue despite the emerging use of analytical methods for fraud detection. This motivates policy makers to also be interested in the provider billing characteristics and understand the common factors that drive conservative and/or aggressive behavior. Statistical approaches to tackling this problem are confronted by the asymmetric and/or leptokurtic distributions of billing data. This paper is a first attempt at using a quantile regression framework and a variable selection approach for medical billing analysis. The proposed method addresses the varying impacts of (potentially different) variables at the different quantiles of the billing aggressiveness distribution. We use the mammography procedure to showcase our analysis and offer recommendations on fraud detection.

## 1. Introduction

The global average health care overpayment loss is estimated to be more than 6 % of overall spending, or more than $450 billion. In the U.S., national health care spending reached $3.6 trillion in 2018, or $11,172 per person [1]. The Federal Bureau of Investigation estimates that up to ten percent of annual health care spending is lost to fraud, waste and abuse. Despite the increasing attention and resources for fraud detection, the extent of health care fraud remains an issue. For instance, in Florida, in 2012 a whistle blower exposed billing fraud in the millions of dollars at seven major hospitals (https://www.radiologybusiness.com/topics/business-intelligence/whistleblower-saw-imaging-billing-fraud-7-florida-hospitals, accessed on 13 August 2019). In 2016, a $1 billion alleged Medicare fraud, money laundering scheme, led to many arrests after its detection, using analytical methods (https://www.cnbc.com/2016/07/22/1-billion-alleged-medicare-fraud-money-laundering-scheme-leads-to-florida-arrests.html, accessed on 13 August 2019). In September 2020, nineteen more defendants were charged in Florida as part of a national crackdown on fraudulent submissions to private insurers and federal health programs to the tune of $6 billion (https://www.justice.gov/usao-mdfl/pr/national-health-care-fraud-and-opioid-takedown-results-largest-enforcement-action, accessed on 13 August 2019).

Health care overpayments are generally classified into three categories—provider, patient and insurer. Among these, overpayments as a result of provider activities, involving fraudulent protocols by hospitals and physicians, are the most common. Examples include, but are not limited to, the submission of false claims, kickback payments, self-referrals, etc.; see [2] for a comprehensive discussion. This paper focuses on upcoding, which is also referred to as overcharging or aggressive billing. Examples of upcoding include the following: (a) exaggerating the time to perform a procedure; (b) deliberately mis-specifying the equipment needed in the procedure; (c) misrepresenting the credentials of the staff performing the procedure in order to increase payment rates; and (d) lying that the procedure happened when in fact it did not.

Upcoding is a concern for both private and government insurance programs. The most straightforward way to detect potential upcoding is through manual audits by using the medical charts. However, the size and complexity make comprehensive audits challenging, if not impossible [3]. Therefore, statistical methods are widely used to detect fraudulent transactions; see [4,5,6] for overviews. Standard practice of upcoding detection typically uses outlier detection to isolate providers with more aggressive billing patterns as potential audit targets. For any procedure and/or provider, one could compare suspicious billings with historical claims data. For instance, Silverman and Skinner [7] show that profit driven hospitals biased their claims toward higher-paying diagnoses to maximize reimbursement while [8] discuss cases of upcoding at non-profit hospitals. However, as [9] noted, identifying upcoding in claims data is difficult due to confounding variables such as patient risk. Kc and Terwiesch [10] argue that patient selection bias is a potential issue in such studies. Indeed, Ata et al. [11] show that payment models could offer incentives to hospitals to admit short-lived patients. Particular payment plan characteristics could also motivate providers toward aggressive billing. Brunt [12] show that drastic changes in the Medicare fee differentials for similar procedures increase the likelihood of upcoding. Zafari and Ekin [13] and Ekin et al. [14] propose a combination of Bayesian hierarchical and outlier detection approaches to group and detect unusual provider billings. Zafari et al. [15] present an integrated multivariate outlier detection and decision modeling framework.

While identification is crucial and helps with recovery of overpayments, it does not change the overall culture of upcoding; hence, long term system integrity is compromised. This motivates policy makers to be interested in the provider billing characteristics as well as understanding the common factors that drive conservative and/or aggressive behavior. For instance, Cooper et al. [16] point out the low correlation between the growth in hospital referral region-level private health spending and growth in fee-for-service Medicare spending; they argue that different factors may be driving the growth in spending for different systems. This study focuses on U.S. Medicare fee for services systems and uses claims data to study the key factors that can explain differences in billing patterns. Many factors contribute to determination of payment amounts for Medicare Part B fee for services. These include, but are not limited to, the 20% co-payment; non-participating status and limiting charge; facility & non-facility rates; geographic adjustments; and multiple procedure payment reductions. Among these, geographical adjustments are one of the main reasons for payment differences since, typically, urban areas have 5% to 10% higher payment rates than national averages. Medicare allowed payment amounts for each procedure in each geographical region are publicly available at [17]; see, for instance, [18] for a county level analysis. Although these payment amounts are determined with respect to well-specified fixed formulas that account for geographic and billing type variations, the billing patterns among providers may still differ even for the same procedure. For instance, underlying common factors for providers that request higher reimbursement for same set of services could be different than the providers with conservative billing behaviors. Even if the factors are similar, their marginal impact could be different. Understanding such differences in billing patterns could enable policy makers offer specific policies. This, in turn, can also lead to better upcoding detection by decreasing the amount of false positives.

A variety of statistical approaches are proposed to study the common factors underlying billing patterns, see [19] for a discussion. Linear regression models are often used. Jürges and Köberlein [20] use regression to determine relationships between upcoded cases and expected payment differences; Bowblis and Brunt [21] analyze the impact of geographic adjustments on profit margins; Fang and Gong [22] attempt to detect potential overbilling in Medicare reimbursement. Such oft-use of linear models is somewhat surprising, given the complexity of the data. Indeed, since billing data distributions are asymmetric and/or leptokurtic, the use of ordinary least square regression could lead to serious biases in fraud detection. A limited number of hybrid studies use regression methods along with unsupervised methods [23]. Lorence and Spink [24] analyze survey-based variations in billing patterns via descriptive analytics. A logistic Bayesian model is developed to detect questionable claims and estimate probability of upcoding [25].

We introduce quantile regression models to the domain of health care fraud detection. The use of quantile regression in health care utilization and fraud assessment has been limited. The only relevant quantile regression based application examines health care expenditures during the Great Recession [26]. To the best of our knowledge, our paper is the first study that investigates the underlying characteristics of both aggressive and conservative billing by providers in health care systems via quantile regression and variable selection methods. Under this approach, providers at lower quantiles of the aggressiveness distribution could be argued to be more conservative or efficient, or even potentially underpaid. Their efficient behavior can serve as a benchmark for other providers. On the other hand, providers at the higher quantiles of the aggressiveness distribution could be upcoding the system. Characteristics unique to this latter group may help improve our understanding of their billing behavior. A related issue is the set of covariates that could influence aggressive billing could be quite large and may have differing impacts on the data distribution’s quantiles. We propose using the Bayesian Information Criterion (BIC) to select key variables at several quantiles of the billing distribution. This quantile variable selection framework is a first in the statistical modeling of fraud detection.

In order to conduct the empirical analysis; in addition to using the fee for service payment data from Medicare Part B, we construct a large database by combining a number of public data sources. With these data, for the mammography procedure, we find that the variables which impact the various quantiles of the billing aggressiveness distribution are very different than those obtained from a linear regression. Moreover, the set of variables differ across the various quantiles of the billing distribution. We discuss how our findings can be used in health care fraud prevention and detection. The paper proceeds as follows. Section 2 discusses the data, quantile regression and variable selection methodologies. This is followed by an empirical analysis in Section 3. A discussion and an overview in Section 4 and Section 5 conclude the paper.

## 2. Materials and Methods

### 2.1. Data

*Data Sources.* The major data set of use is the *The Physician and Other Supplier Public Use File* [27], which contains 100% final-action physician Medicare Part B services and procedures provided to fee for service (FFS) beneficiaries. It includes measurements for utilization, payment and submitted charges organized by the *National provider identifier* (NPI), *Healthcare common procedure coding system* (HCPCS) code, and place of service. We chose the year 2014 to take into account the major payment reduction adjustment after April 1, 2013, and the addition of the variable “standardized payment”. For fair comparison, we only use observations for individual entities that have facility as place of services.

The raw health care claims data has 9,316,307 observations in which each row corresponds to the billing summary of a given procedure by a particular provider. It is straightforward to use our approach for data of any procedure, as well as any given provider or provider-procedure pairs. We focus on the fee for service claim billings for Procedure 77055, which corresponds to computer-aided detection applied to a diagnostic mammogram, in order to illustrate the proposed method. Following the recent Affordable Care Act, starting 1 January 2020, mammography was reclassified as a preventive, not diagnostic, screening procedure. Developing a dataset for the analysis of fraudulent billing behavior is a formidable challenge due to the complexity and size of the source databases. In the following, we describe how we accomplished this task in general, keeping in mind we used the process to isolate the upcoding practice for Procedure 77055.

The characteristics of a given procedure-provider pair (with a unique HCPCS service code and a unique NPI) are the focal variables. These include the average Medicare allowed payment amount (the Medicare payment, deductible and copay averaged over procedure); average submitted charge amount; average Medicare payment amount after deduction of deductible and coinsurance; and average Medicare standardized payment amount that removes geographical differences in payment rates. The claims data also include number of unique beneficiaries served; number of services provided; and number of distinct Medicare beneficiary/per day services. Provider-specific information, such as the state, zip-code, address and provider type, are also used. In order to measure the impact of provider type for claims on a given procedure, we computed the mean and standard deviation of the aggressiveness ratios that capture the relative frequency and deviation.

Next utilized data source is the *Fee Schedules* to map the zip codes to locality information [28]. The current *Physician Fee Schedule* locality structure was developed and implemented in 1997. At present, there are 89 localities of which 34 are statewide areas,. which account for the geographical variation in payment patterns; see [29] for details. Then, we extract the geographical practice cost indices for each locality [17]. In particular, the relative costs of physician work, practice expense, and malpractice expense in a specific area, compared to the national average costs, for each component are retrieved; see [30] for details. Lastly, we employ a public use file that provides demographic, spending, utilization, and quality indicators at the state level. This can help evaluate geographic variation in the utilization and quality of health care services for the Medicare fee for service population; see [31] for details.

**Figure 1:**
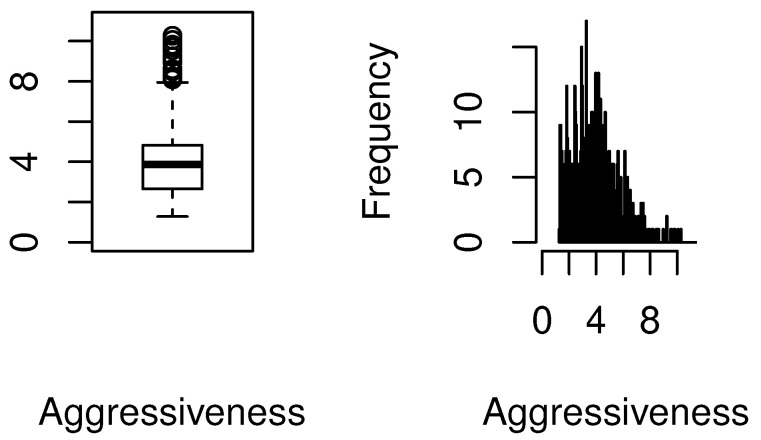
Boxplot and histogram of provider billing aggressiveness for Procedure 77055.

*Response variable.* “Billing aggressiveness of the provider” for the mammography procedure is defined as a non-negative measurement for which larger values potentially reflect upcoding for the same procedure. It is the ratio of the average submitted charged amount and average payment amount for all claims submitted for a given procedure [9]. The first row of Table 1 displays the skewness of the standardized payments for Procedure 77055. There are a total of 685 providers that billed for this procedure. It can be seen that the standard deviation of standardized payments is approximately ten percent of the mean amount for all providers. The skewness and kurtosis values are high. Per our earlier discussions, such discrepancies in the standardized average payment amounts could be explained by geographical variations and patient related factors. However, we should emphasize that our focus is not on payments per se, rather our analysis aims to investigate the potential reasons for differences in the aggressiveness of providers for the same procedure. The second row of Table 1 exhibits the right skew in the distribution of the aggressiveness of the providers. Both skewness and kurtosis values are very high. The skewness and the existence of outliers are also displayed in the box-plot and histogram of billing aggressiveness; see Figure 1. The table and graphs point to the non-normality of the billing distribution for this procedure.

*Covariates.* We consider the following covariates.

- Line Service Count: This corresponds to the number of services provided by the particular provider of interest for that procedure.
- Unique Beneficiary Count: This is the number of distinct Medicare beneficiaries receiving the service. This is expected to be related to the *Line Service Count*, however it can provide additional insights especially for fraudulent and/or aggressive provider billing behavior.
- Average Medicare Allowed Payment: This is the sum of payment amount by Medicare, the deductible and coinsurance amounts paid by the beneficiary, and any amounts that a third party is responsible for paying.
- Average Medicare Standardized Payment: This variable captures the average standardized payment amount by Medicare after the deduction of beneficiary deductible and coinsurance amounts and geographical standardization. Standardization removes geographic differences in payment rates for individual services due to local prices and makes Medicare payments across geographic areas comparable. This allows to reflect variations and capture underlying factors of providers’ billing patterns [32].
- GPCI Work: The Geographical Practice Cost Index (GPCI) has been established for every Medicare payment locality for each of the three components of a procedure’s relative value unit (RVU) for work, practice expense, and malpractice. Since these are highly correlated, we picked the RVU for work.
- MA Participation Rate: These are state rates for the Medicare Advantage (MA) participation. This is chosen since it can provide a glimpse of the billing patterns within that state, in particular of the aggressiveness of provider billing.
- Average Age: This is the numerical variable of age, which is mostly above 65 given the nature of Medicare program. In general, the older patients are taken more advantage of, so this can help understand billing patterns.
- Percent Female: This is coded as a numerical variable that provides the ratio of female out of all beneficiaries. We aim to investigate if the gender of the beneficiaries has an association with billing patterns.
- Percent non-Hispanic White: We aim to investigate if the racial demographic of the beneficiaries has an association with billing patterns.
- Percent Eligible for Medicaid: This is the state rate for the percent of Medicare beneficiaries that are eligible for Medicaid. Dual Medicare-Medicaid (Medi-Medi) access are available to those who meet certain criteria. This has been an area of upcoding, and hence motivated the initation of Medi-Medi Data Match Program [33].
- Average HCC Score: The Hierarchical Condition Category (HCC) score is a risk-adjusted value that ranks diagnoses into categories which represent conditions with similar cost patterns. Higher categories represent higher predicted health care costs, resulting in higher risk scores. Average HCC Score represents the averaqe risk score for Medicare beneficiaries of all ages in each region. The national average is 1.0.
- Standardized Risk-adjusted Per Capita Costs: This corresponds to the cost of Medicare procedures adjusted to reflect all beneficiaries for each state.
- Mean Aggressiveness of Provider Type: This is defined as the average ratio of the average submitted charged amount and average payment amount for that particular provider type. Billing for certain procedures are expected to be more aggressive for some provider types, so we aim to capture that.
- Standard Deviation of Aggressiveness of Provider Type: This is defined as the standard deviation of the average submitted charged amount and average payment amount for that particular provider type. Some provider types could include various distinct types of provider billing patterns, so this variable aims to help capture that volatility. It can be expected that a provider with a type that has low standard deviation of aggressiveness, would behave within billing norms associated with that provider type.

The asymmetric and/or leptokurtic distributions of billing data need to be considered while modeling the the underlying characteristics of provider billing patterns. Our first hypothesis is that different quantiles of the distribution of billing aggressiveness may be affected by different covariates. Our second hypothesis is that the magnitude of the impact of the covariates differ at quantiles of the distribution of billing aggressiveness. In order to check for those hypotheses, we use the statistical methods of quantile regression and quantile variable selection, which are described in the following.

### 2.2. Quantile Regression

A practical option for upcoding detection is to flag any provider with aggressiveness higher than a certain threshold as an outlier for audits. However, in order to ensure program integrity, we also need to understand the underlying characteristics of aggressive providers and upcoding. Health care billing is a complex phenomena, and causality is difficult to attain. We emphasize that we do not claim causal relationships between the response and independent variables in our model. A major limitation of existing classical linear regression models is their inability to quantify the differing impacts of explanatory variables on different parts of the data distribution. They might provide acceptable explanatory power “on average” but may be poor at modeling abnormal swings. Consider Table 1. The factors that impact the lower quantiles of the aggressiveness distribution may be different than those at the upper quantiles. And even if the factors coincide, their marginal impacts at the lower and upper quantiles could be different. A change in the “payment amount” may have little impact if billing aggressiveness is low, but it could result in a great impact in billing aggressiveness in case it is already high. These varying relationships, and differing impacts of variables, on different levels of aggressiveness cannot be captured by mean linear regression models. For settings with many variables, a variable selection method can be valuable to identify the best possible subset of covariates in the quantile regression model. In addition to handling non-normal data distributions, the contextual relevance of understanding what covariates influence the entire data distribution motivates us to employ quantile regression variable selection techniques.

Following [34,35,36,37], the linear quantile regression model for the *i*th observation of the response variable *y* is given by:

(1) $$y_{i}=X_{i}^{T}{\mathrm{fi}}^{*}+e_{i}\;\;i=1,\cdots ,n,$$

where the error terms, $e_{i}$ are i.i.d; P(e≤0|X=x)=τ for almost every independent variable x; $X_{i}={\left(X_{i1},\cdots ,X_{ip}\right)}^{T}$; and ${\mathrm{fi}}^{*}={\left(\beta_{1}^{*},\cdots ,\beta_{p}^{*}\right)}^{T}$.

Given that our aggressive billing data is highly asymmetric and heavy-tailed, following [38], we assume that each $e_{i}$ follows an asymmetric Laplace distribution with density function

(2) $$f\left(e\right)=\tau \left(1-\tau \right)\sigma^{-1}exp\left(-\rho_{\tau }\left(e\right){\left(2\sigma \right)}^{-1}\right),$$

where $\rho_{\tau }\left(e\right)=e\left(2\tau -2I\left(e<0\right)\right)$, $e_{i}$ is independent of $X_{i}$, and *I* is the indicator function. Thus, the conditional τ-quantile of $y_{i}$ given $X_{i}=x_{i}$ is ${x_{i}}^{T}{\mathrm{fi}}^{*}$.

### 2.3. Quantile Variable Selection

The Bayesian Information Criterion (BIC) method [39] is known to provide best subset selection as it identifies the true model consistently [40]. Modified forms of the BIC to account for large model spaces and large sample sizes are presented to have sound statistical properties [41,42]. These improvements to BIC are based on embedding robust shrinkage estimation methods such as the least absolute shrinkage and selection operator (LASSO) and smoothly clipped absolute deviation (SCAD); see [38,43].

The aim is to consider only $d^{*}$ independent variables among the $X_{ij}$s, implying $p-d^{*}$ covariates to be set as zero in Equation (1). Adapting the notation from [38], let $S=\left\{j_{1}\cdots ,j_{d}\right\}\subset \left\{1,\cdots ,p\right\}$ denote a candidate model corresponding to the independent variables $X_{j1},\cdots ,X_{jd}$: denote $X_{s}={\left(X_{j1},\cdots ,X_{jd}\right)}^{T}$; |S| to be the cardinality *d* of *S*; $\left({\hat{\beta }}_{S},\hat{\sigma }\right)$ to be the maximum likelihood estimator of $\left(\beta_{S},\sigma \right)$. Then, the BIC for linear quantile regression is given by:

(3) $$\mathrm{BIC}\left(S\right)=log\left(\Sigma_{i=1}^{n}\rho_{\tau }\left(y_{i}-X_{iS}^{T}{\hat{\beta }}_{S}\right)\right)+\left|S\right|log\;n{\left(2n\right)}^{-1}C_{n},$$

where $C_{n}$ is a positive constant that diverges to infinity as *n* increases. A discussion related to the theoretical properties of Equation (3) can be found in [38]; these authors also demonstrate how the BIC successfully identifies the true model in high-dimensional quantile regression models.

Let ${\hat{\beta }}_{\lambda }={\left({\hat{\beta }}_{\lambda ,1},\cdots ,{\hat{\beta }}_{\lambda ,p}\right)}^{T}$. The process of selecting the best subset model proceeds by choosing λ>0 as follows:

(4) $$\hat{\lambda }=\underset{\lambda }{\arg\min}(log\left(\Sigma_{i=1}^{n}\rho_{\tau }\left(y_{i}-X_{iS}^{T}{\hat{\beta }}_{\lambda }\right)\right))+\left|{\hat{S}}_{\lambda }\right|log\;n{\left(2n\right)}^{-1}C_{n}.$$

The selected subset ${\hat{S}}_{\lambda }\equiv \left\{j:{\hat{\beta }}_{\lambda ,j}\neq 0,\;1\leq j\leq p\right\}$ includes the variables that are selected at the τth quantile of the distribution of the dependent variable *y*. In order to find the estimators for ${\hat{\beta }}_{\lambda }$, we use the LASSO procedure in R [44]. We let λ vary between 0.01 and 1. If a particular independent variable’s coefficient estimate is less than 0.001, that is set to zero, which means it is not selected in the model. For the optimal λ, the module presents the corresponding estimates for ${\hat{\beta }}_{\lambda }$ and the BIC values at each of the selected quantiles. The following quantiles, τ, are selected: 0.01, 0.05, 0.25, 0.50, 0.75, 0.95 and 0.99; collectively, these quantiles better capture the impact of the covariates at various positions along the entire distribution of aggressiveness; importantly, the impact at the tails of the aggressive billing distribution are also accounted for in the process.

## 3. Results

We first estimate the parameters corresponding to the covariates via the OLS mean regression model which could be used in fraud detection. We then employ the proposed methodology on the same data and variables.

### 3.1. OLS Estimation

Consider Table 2. Average Medicare standardized payment amount and mean aggressiveness of the provider type are significant factors. Most of the providers that are billing for this procedure are diagnostic radiologists; this is crucial in understanding upcoding. For instance, the average aggressiveness of general surgeons is 6.06, which is higher than the overall mean of 4.00. The billing pattern of a general surgeon is likely to be more aggressive than average. Another finding is that providers exhibit higher aggressiveness when the amount of average standardized payment is lower (*p* = 0.000). Lastly, it should be noted that an increase in average HCC score, which indicates aggregate sickness level of patients, also increases billing aggressiveness (*p* = 0.082).

**Figure 2:**
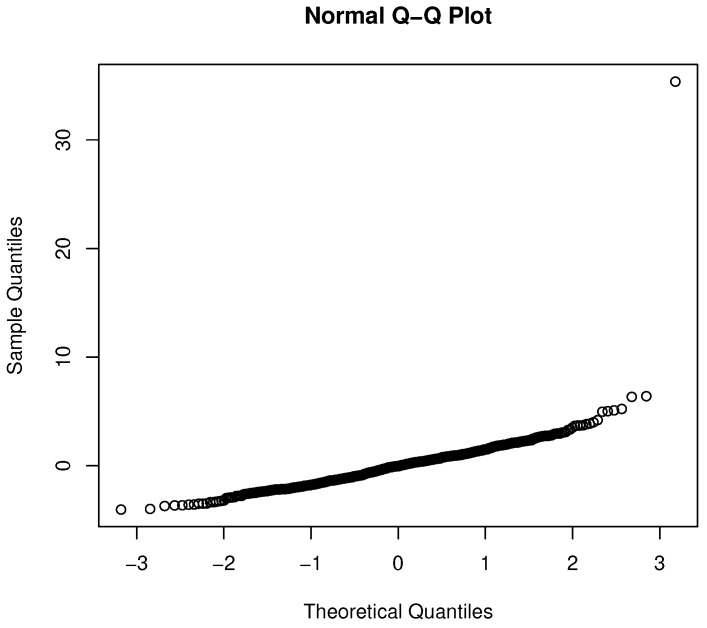
Q-Q plot for the OLS regression.

However, not surprisingly, the residuals from the OLS analysis violate the normality and iid assumptions. The Lilliefors test for non-normality is significant (*p* = 2.133 × 10${}^{-13}$), which is also evidenced in the q-q plot shown in Figure 2. In short, the analysis shown in Table 2 could lead to a poor understanding of providers that overcharge.

### 3.2. Quantile Estimation

Setting aside variable selection for the moment, consider Table 3 which presents the quantile regression coefficients and their significance levels at the quantiles {0.01, 0.05, 0.25, 0.5, 0.75, 0.95, 0.99}. The varying impacts of variables at the different quantiles of the aggressive billing distribution are evident. While average Medicare standardized payment amount is significant for every quantile of interest, please note that the mean aggressiveness of the provider type is only significant for three quantiles. Let us examine just a few of these relationships below in the interests of brevity.

First, the average Medicare standardized payment amount is significant for all quantiles, albeit with varying beta coefficients at different quantiles. For higher percentiles of aggressiveness, the relationship becomes stronger and more negative. The providers with increasing aggressive billing patterns become less aggressive for higher standardized payment amounts. Second, average HCC score, has a positive impact on aggressive billing, but is only significant at percentiles of 0.05 and 0.95. Similarly, the variable *GPCI Work*, which measures the geographic variation adjustment of practice expense, is only significant at 25th percentile. Third, consider the impact of standard deviation of aggressiveness for each provider type. This variable’s marginal effect trends from negative to positive with increasing values. Fourth, upcoding tends to be higher among less aggressive providers that bill in states where patient populations have higher proportions of dual eligibility—those that are eligible for Medicaid and Medicare. This relationship becomes inverse for providers with increasing aggressiveness. Upcoding is also higher in states with more non-Hispanic, white demographic patients; although this is not significant.

**Figure 3:**
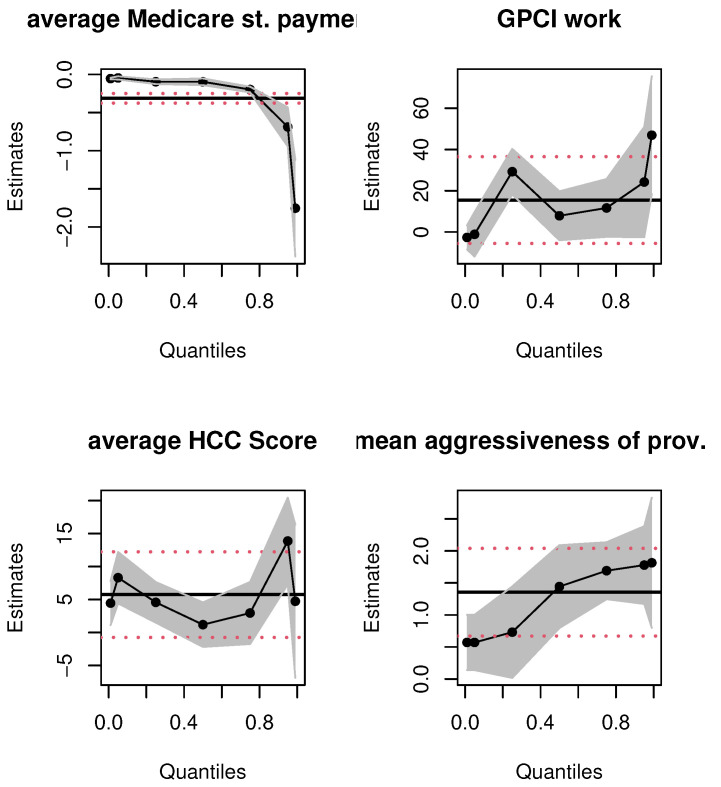
OLS and quantile regression estimates for select variables. Notes: *x*-axis depicts quantiles of the dependent variable; *y*-axis represents parameter estimates. The solid black line and dashed red lines are the OLS and 95% confidence interval estimates, the dotted black line and gray shaded areas are the quantile functional and 95% confidence band estimates, respectively.

To further demonstrate the value of quantile regressions in this context, we graphically compare the confidence intervals and the point estimates given in Table 2 and Table 3. Consider Figure 3 that presents the parameter estimates for the variables “*average Medicare standardized payment amount*”, “*GPCI Work*”, “*average HCC score*”, and “*mean aggressiveness of provider type*”.

The first takeaway is the *non-linear*, non-constant nature of the relationship between the covariates and aggressive billing data; the *linear* OLS estimation misses these true relationships entirely. Second, at first glance, it would seem that since some quantile estimates for each of the covariates are contained in the corresponding OLS-based 95% confidence intervals, there is no major difference in the OLS and quantile estimates. This inference would be misguided. The 95% OLS-based interval is *always* equidistant from the OLS *mean* estimate of a parameter. The quantile estimates are *localized* regression values. To illustrate this, consider the top right panel for the GPCI Work estimates. Most of the quantile estimates are within the 95 % confidence interval of the OLS estimate but consider, for instance, the quantile regression point estimate of GPCI Work at the first quartile. The *localized* marginal effect of GPCI Work on the billing data that fall at this quantile is twice as large as the OLS mean effect; the latter’s effect on billing data is *always*, on average, 15.49 from Table 2, no matter where the data are on the billing distribution. Practically speaking, the impact of the relative cost of physicians’ work is, in fact, twice as large at the lower spectrum of the aggressive billing distribution; the OLS estimate grossly underestimates the impact of this covariate on a sizable number of billing data. A similar argument can be made for average HCC score. Thus, even if the quantile estimates are within the equal-tailed 95% confidence interval of the OLS estimate, they vary significantly and non-linearly across the billing data distribution. Third, consider the top left panel for the average Medicare standardized payment amount; see Table 3 for the corresponding numerical values that quantify the varying nature of the marginal effect of this covariate. In contrast, the linear mean effect for this covariate is fixed throughout the distribution of aggressiveness. The numerical estimates only intersect slightly at the upper 95 % confidence interval of the 75th percentile. This is also evident with the impact of “mean aggressiveness of the provider type”. Although this mean effect is significant for OLS regression, for all quantiles of the quantile regression method, its estimates vary across quantiles, and some are not significant. Finally, the non-linear quantile interval estimates provide evidence of changing standard errors at the different quantiles, but in the OLS estimation the standard error is always fixed; this is implausible given the skew in the billing data distribution shown in Table 1 as well as the plots in Figure 1.

Similar results hold for the other covariates; for brevity, we only present the plots for the variables discussed above. These results validate our key empirical claim that mean regressions are ill-suited to explore the impact of covariates on the billing aggressiveness data as a whole.

### 3.3. Variable Selection

Our hypothesis is that different quantiles of the distribution of billing aggressiveness may be affected by different covariates. To validate this, we investigate which of the 14 covariates are selected by the BIC procedure at each of the quantiles (τ = 0.01, 0.05, 0.25, 0.50, 0.75, 0.95, 0.99).

Table 4 presents the Maximum Likelihood Estimates (MLEs) for the selected variables in the LASSO quantile regression, which quantifies the marginal effects of each variable. As expected, average standardized payment amount (avg. std. payment amount) is kept in each model. The coefficients for avg. std. payment amount display a negative relationship with aggressiveness, which gets stronger for increasing quantiles. The directions of the relationships for other MLE estimates are also in line with previous results. The MA participation rate is kept in models at five quantiles, while its value is larger for higher quantiles. The mean aggressiveness of provider type (mean agg PT) is significant and positive with increasing values for percentiles starting at 0.50. The average allowed payment amount is always positively related to aggressiveness. The marginal effects of the “GPCI Work”, “percent efmal” “services provided” (line service count) vary among quantiles where they are significant. These findings emphasize the advantages of using Quantile Regression versus OLS results in Table 2.

## 4. Discussion

The primary impact of our study is to enable auditors understand billing behavior more accurately by an improved siphoning of providers of each medical procedure. Consider the OLS estimate for “standardized payment amount”. While this variable is negatively (and significantly) associated with billing aggressiveness as displayed by both OLS and quantile regression outputs; the amount of its effect varies over the quantiles of billing distribution. A unit change in the average standardized payment amount has more than 30 times impact for highly aggressive billing data compared to very low aggressiveness. Using OLS, an auditor will not be able to focus on providers accordingly considering their billing aggressiveness; the focus would be on the impact on mean aggressiveness. The second major impact is that mean aggressiveness of the provider type is not significant for quantiles corresponding to conservatively billing providers. When the auditor uses OLS; the assumption is that the whole billing distribution is impacted. Similar to the other variables, the magnitude of the impact also varies. These could result in a large number of false positives. Based on the BIC selection, we can also glean insights about very conservative providers. When the standardized payment amount for the procedure is high, its impact on aggressiveness is lower at at low quantiles. Increasing standardized payment amount have a higher negative impact at more aggressive providers. In other words, the most conservative providers, in terms of billing impact, affect provider payment systems differently. This conclusion is glaringly absent in the OLS mean regression approach.

The analysis also points to potential differences on the impact of covariates on high quantile payment amounts versus median and low quantile payment amounts. While the significant covariates for highly aggressive providers may include mean aggressiveness of provider type, average HCC score and percent of female beneficiaries; MA participation rate is significant for low quantiles.

When we analyze the most aggressive individual providers, we would typically merely red flag the top providers for audit, number depending on our resources. But now, our analysis can include the crucial insight of the (statistically significant) patients with low average HCC score offered by this provider when compared to the rest of the providers at that quantile. Moreover, this provider may be providing services in a state with high percent of female beneficiaries. In conjunction with the mean aggressiveness values among that provider type (diagnostic radiologists), such information could be used as potential clues to detect cases of upcoding.

## 5. Conclusions

The proposed quantile regression and variable selection framework is utilized on a combined CMS Medicare data set to determine the key factors that reveal billing aggressiveness patterns with respect to the geography, provider and procedure type. It can also help with potential audits of suspicious providers, decrease the amount of false positives, and identify the underlying determinants of upcoding.

We used data from Medicare Part B, which is a fee for service (FFS) payment system. In FFS systems, the insurer reimburses the provider for every procedure or test done on the patient. CMS has been actively experimenting with novel payment models. For instance, the alternative of bundled payments are utilized in programs such as Medicare Advantage: these plans receive a fixed monthly payment-per-beneficiary to cover potential health care costs. They are incentivized to improve the patients’ health at minimum cost. With these policy changes and novel experiments, it will be even more important to understand the billing patterns of providers in payment systems. It could be worthwhile to extend our methodology to other programs’ payment data. For instance, Medicare Advantage programs employ risk adjustment payment systems. These also have cases of upcoding; see [45].

Our approach can also be extended for a type-procedure pair to see the differences among billing relationships. For instance, we can take a subset of all cardiologists who billed for a particular procedure (say 99213—office outpatient visit). The skewness for such billings and non-linear nature of the varying impacts of the covariates can be captured with the proposed methods.

One of the main limitations of our paper is the lack of patient-specific characteristics and data for quality of care. Although we tried to capture that impact by using average HCC scores, it is still only at an aggregate level and may not reflect a patient’s well-being. In order to capture the impact of quality of care, CMS has established a budget-neutral “value modifier”. This measure provides differential payments to providers based on the quality of care compared to the cost of care furnished to Medicare FFS beneficiaries during a performance period. Incorporating that to our analysis can improve the results.

## Figures and Tables

**Table 1:** Summary statistics for *Average Standardized (Std) Payment Amounts* and *Aggressiveness* for Procedure 77055.

Variable	Mean	SD	Skewness	Kurtosis	1stP	5thP	25thP	50thP	75thP	95thP	99thP
Std. Payment	25.58	2.61	−1.79	9.67	17.19	21.07	24.13	26.12	27.88	28.09	28.52
Aggressiveness	4.00	2.32	8.91	156.52	1.34	1.53	2.65	3.86	4.82	6.83	9.29

**Table 2:** OLS regression statistics where billing *aggressiveness* for Procedure 77055 is the dependent variable.

Variable Name	Estimate	s.e.	*p*-Value
(Intercept)	4.845	17.389	0.781
line service count	−0.009	0.045	0.847
unique beneficiary count	0.006	0.047	0.906
average Medicare allowed payment amount	0.130	0.085	0.126
average Medicare standardized payment amount	−0.312	0.032	**0.000**
GPCI Work	15.490	10.712	0.149
MA participation rate	0.986	1.284	0.443
average age	−0.243	0.231	0.293
percent female	−13.678	13.174	0.300
percent non-Hispanic white	1.489	1.351	0.271
percent eligible for Medicaid	−2.916	3.648	0.424
average HCC Score	5.749	3.306	0.082
standardized risk-adjusted per capita costs	0.000	0.000	0.554
mean aggressiveness of provider type	1.356	0.349	**0.000**
std. dev. of aggressiveness of provider type	−0.159	0.862	0.854

**Table 3:** Quantile regression coefficient estimates for each variable for the percentiles of {0.01, 0.05, 0.25, 0.5, 0.75, 0.95, 0.99} (Significance: [0, 0.001): ***, [0.001, 0.01): **, [0.01, 0.05): *).

Variable/Quantiles	0.01	0.05	0.25	0.50	0.75	0.95	0.99
Intercept	14.615	21.606	2.269	12.837	19.458	35.301	38.981
line service count	−0.024	−0.037	−0.002	−0.005	−0.018	0.047	0.029
unique beneficiary count	0.028	0.043	0.001	0.006	0.015	−0.062	−0.049
average Medicare allowed payment amount	0.018	0.019	0.1	0.168	0.156	0.035	−0.258
average Medicare standardized payment amount	**−0.054** ***	**−0.045** *	**−0.095** **	**−0.096** *	**−0.197** ***	**−0.688** **	**−1.754** **
GPCI Work	−2.646	−1.136	**29.248** **	7.858	11.645	24.215	46.94
MA participation rate	−1.713	**−3.604** *	−0.499	1.945	2.477	−1.062	3.801
average age	−0.102	−0.161	**−0.461** *	−0.362	**−0.443** *	−0.432	−0.559
percent female	−13.551	−21.868	−7.706	−0.228	−9.378	**−49.686** *	3.091
percent non-Hispanic white	0.751	−0.044	**2.131** *	0.233	1.069	2.368	2.816
percent eligible for Medicaid	3.275	0.23	−1.798	−2.479	−2.919	−3.881	−4.46
average HCC score	4.459	**8.314** *	4.577	1.194	2.958	**13.875** *	4.741
standardized risk-adjusted per capita costs	0	0	0	0	0	0	0
mean aggressiveness of provider type	0.571	0.57	0.731	**1.443** *	**1.691** ***	**1.779** **	1.815
std. dev. of aggressiveness of provider type	−1.168	−0.922	0.007	−0.065	0.234	1.54	1.775

**Table 4:** MLE’s for chosen variables for LASSO Quantile Regression (Note: All the other variables are not selected, and hence have MLEs of 0).

Variables/Quantiles	0.01	0.05	0.25	0.5	0.75	0.95	0.99
(Intercept)	1.765	2.438	−1.424	2.547	0.921	−0.114	26.903
line service count	0	0	0	0	−0.002	0	0
avg. allowed payment amount	0.005	0	0.139	0.135	0.103	0.156	0
avg. std. payment amount	−0.019	−0.032	−0.032	−0.053	−0.136	−0.160	−0.834
GPCI Work	0	0	0	0	3.125	8.870	0
MA participation rate	−0.192	−0.121	0	0.447	0.546	0.156	0
percent female	0	0	0	−5.091	−6.508	0	0
mean agg PT	0	0	0	0.145	1.043	0.509	0.765

## Data Availability

Data are available from CMS (https://data.cms.gov/, accessed on 13 August 2021).
